# Supplementary material for: Multi‐dataset identification of innovative feature genes and molecular mechanisms in keratoconus
Source: J Cell Mol Med. 2024 Sep 19;28(18):e70079. doi: 10.1111/jcmm.70079 (PMC11412914; doi:10.1111/jcmm.70079)
Supplement: Supplementary file 2 — Material S2. [file JCMM-28-e70079-s002.zip › 7.GSEA analysis/6.SNORA74B/KEGG_TGF_BETA_SIGNALING_PATHWAY.html]

Details for gene set KEGG\_TGF\_BETA\_SIGNALING\_PATHWAY[GSEA]

|  || Dataset | SNORA74B.group.cls#h\_versus\_l |
| Phenotype | group.cls#h\_versus\_l |
| Upregulated in class | l |
| GeneSet | KEGG\_TGF\_BETA\_SIGNALING\_PATHWAY |
| Enrichment Score (ES) | -0.4354463 |
| Normalized Enrichment Score (NES) | -1.4720019 |
| Nominal p-value | 0.044145875 |
| FDR q-value | 0.3422343 |
| FWER p-Value | 0.821 |
Table: GSEA Results Summary

  

Fig 1: Enrichment plot: KEGG\_TGF\_BETA\_SIGNALING\_PATHWAY      
 Profile of the Running ES Score & Positions of GeneSet Members on the Rank Ordered List

  

| SYMBOL | TITLE | RANK IN GENE LIST | RANK METRIC SCORE | RUNNING ES | CORE ENRICHMENT || 1 | GDF6 | na | 1861 | 0.384 | -0.0255 | No |
| 2 | BMPR1B | na | 2240 | 0.352 | -0.0105 | No |
| 3 | AMHR2 | na | 2619 | 0.325 | 0.0025 | No |
| 4 | NOG | na | 2881 | 0.308 | 0.0176 | No |
| 5 | BMP8B | na | 3989 | 0.260 | 0.0048 | No |
| 6 | GDF7 | na | 5124 | 0.221 | -0.0117 | No |
| 7 | NODAL | na | 5852 | 0.197 | -0.0182 | No |
| 8 | ZFYVE9 | na | 8189 | 0.132 | -0.0759 | No |
| 9 | ROCK1 | na | 8851 | 0.117 | -0.0864 | No |
| 10 | ACVR2B | na | 9239 | 0.108 | -0.0896 | No |
| 11 | TGFBR2 | na | 9513 | 0.102 | -0.0899 | No |
| 12 | CDKN2B | na | 10073 | 0.091 | -0.0994 | No |
| 13 | BMPR2 | na | 10089 | 0.091 | -0.0931 | No |
| 14 | SMAD5 | na | 10496 | 0.083 | -0.0987 | No |
| 15 | CREBBP | na | 10525 | 0.083 | -0.0934 | No |
| 16 | INHBC | na | 10667 | 0.080 | -0.0916 | No |
| 17 | ZFYVE16 | na | 11396 | 0.069 | -0.1076 | No |
| 18 | SP1 | na | 11587 | 0.066 | -0.1082 | No |
| 19 | BMP8A | na | 11726 | 0.064 | -0.1075 | No |
| 20 | TGFB2 | na | 11803 | 0.063 | -0.1051 | No |
| 21 | ACVRL1 | na | 11851 | 0.062 | -0.1019 | No |
| 22 | PPP2R1B | na | 12000 | 0.060 | -0.1017 | No |
| 23 | RPS6KB2 | na | 12925 | 0.048 | -0.1249 | No |
| 24 | SMAD4 | na | 13214 | 0.045 | -0.1299 | No |
| 25 | SMAD2 | na | 13222 | 0.045 | -0.1269 | No |
| 26 | THBS3 | na | 13444 | 0.042 | -0.1301 | No |
| 27 | RPS6KB1 | na | 13499 | 0.042 | -0.1286 | No |
| 28 | RBX1 | na | 14044 | 0.035 | -0.1417 | No |
| 29 | ID1 | na | 14640 | 0.028 | -0.1568 | No |
| 30 | THBS1 | na | 14896 | 0.025 | -0.1623 | No |
| 31 | ACVR1C | na | 15522 | 0.019 | -0.1789 | No |
| 32 | CHRD | na | 15880 | 0.016 | -0.1881 | No |
| 33 | SMAD3 | na | 16318 | 0.011 | -0.1999 | No |
| 34 | MAPK3 | na | 16753 | 0.007 | -0.2119 | No |
| 35 | TFDP1 | na | 17332 | 0.001 | -0.2285 | No |
| 36 | ROCK2 | na | 17655 | -0.002 | -0.2376 | No |
| 37 | SMAD1 | na | 17972 | -0.006 | -0.2463 | No |
| 38 | SKP1 | na | 18228 | -0.009 | -0.2530 | No |
| 39 | RHOA | na | 18290 | -0.009 | -0.2541 | No |
| 40 | RBL2 | na | 18808 | -0.015 | -0.2679 | No |
| 41 | BMP5 | na | 20077 | -0.029 | -0.3024 | No |
| 42 | CUL1 | na | 20440 | -0.033 | -0.3104 | No |
| 43 | MAPK1 | na | 20606 | -0.035 | -0.3126 | No |
| 44 | PPP2R1A | na | 21264 | -0.043 | -0.3284 | No |
| 45 | BMPR1A | na | 21644 | -0.047 | -0.3358 | No |
| 46 | LTBP1 | na | 21663 | -0.048 | -0.3328 | No |
| 47 | BMP7 | na | 21836 | -0.050 | -0.3341 | No |
| 48 | RBL1 | na | 21873 | -0.050 | -0.3315 | No |
| 49 | ACVR2A | na | 22361 | -0.056 | -0.3414 | No |
| 50 | LEFTY1 | na | 22851 | -0.063 | -0.3509 | No |
| 51 | SMURF2 | na | 24938 | -0.097 | -0.4039 | No |
| 52 | PPP2CA | na | 25151 | -0.101 | -0.4026 | No |
| 53 | EP300 | na | 25385 | -0.105 | -0.4016 | No |
| 54 | MYC | na | 25534 | -0.108 | -0.3980 | No |
| 55 | SMURF1 | na | 25836 | -0.113 | -0.3983 | No |
| 56 | PPP2CB | na | 26235 | -0.122 | -0.4008 | No |
| 57 | THBS4 | na | 26603 | -0.130 | -0.4019 | No |
| 58 | ID3 | na | 26804 | -0.135 | -0.3977 | No |
| 59 | TGFBR1 | na | 27137 | -0.142 | -0.3968 | No |
| 60 | THBS2 | na | 27307 | -0.147 | -0.3909 | No |
| 61 | PITX2 | na | 28852 | -0.190 | -0.4215 | Yes |
| 62 | E2F4 | na | 29168 | -0.200 | -0.4159 | Yes |
| 63 | ID4 | na | 29192 | -0.200 | -0.4018 | Yes |
| 64 | AMH | na | 29285 | -0.203 | -0.3895 | Yes |
| 65 | ACVR1 | na | 29917 | -0.222 | -0.3914 | Yes |
| 66 | TGFB1 | na | 30144 | -0.231 | -0.3809 | Yes |
| 67 | SMAD7 | na | 30382 | -0.240 | -0.3701 | Yes |
| 68 | E2F5 | na | 30880 | -0.262 | -0.3651 | Yes |
| 69 | INHBE | na | 30990 | -0.268 | -0.3486 | Yes |
| 70 | TGFB3 | na | 31437 | -0.291 | -0.3401 | Yes |
| 71 | LEFTY2 | na | 31467 | -0.293 | -0.3194 | Yes |
| 72 | GDF5 | na | 31574 | -0.299 | -0.3004 | Yes |
| 73 | INHBB | na | 31728 | -0.308 | -0.2822 | Yes |
| 74 | TNF | na | 31738 | -0.308 | -0.2598 | Yes |
| 75 | ID2 | na | 32079 | -0.327 | -0.2455 | Yes |
| 76 | COMP | na | 32169 | -0.332 | -0.2237 | Yes |
| 77 | INHBA | na | 32346 | -0.344 | -0.2035 | Yes |
| 78 | IFNG | na | 32943 | -0.389 | -0.1921 | Yes |
| 79 | BMP6 | na | 33230 | -0.417 | -0.1697 | Yes |
| 80 | SMAD6 | na | 33231 | -0.417 | -0.1390 | Yes |
| 81 | BMP4 | na | 33250 | -0.419 | -0.1088 | Yes |
| 82 | DCN | na | 33278 | -0.422 | -0.0785 | Yes |
| 83 | BMP2 | na | 33393 | -0.437 | -0.0497 | Yes |
| 84 | SMAD9 | na | 33765 | -0.488 | -0.0245 | Yes |
| 85 | FST | na | 34519 | -0.711 | 0.0060 | Yes |
Table: GSEA details [plain text format]

  

Fig 2: KEGG\_TGF\_BETA\_SIGNALING\_PATHWAY      
 Blue-Pink O' Gram in the Space of the Analyzed GeneSet

  

Fig 3: KEGG\_TGF\_BETA\_SIGNALING\_PATHWAY: Random ES distribution      
 Gene set null distribution of ES for **KEGG\_TGF\_BETA\_SIGNALING\_PATHWAY**
